# Supplementary material for: An Ex Vivo Intervertebral Disc Slice Culture Model for Studying Disc Degeneration and Immune Cell Interactions
Source: Cells. 2025 Aug 8;14(16):1230. doi: 10.3390/cells14161230 (PMC12384586; doi:10.3390/cells14161230)
Supplement: Supplementary file 1 [file cells-14-01230-s001.zip › cells-3765704-supplementary.pdf]

Supplemental Materials

Additional Methods

Macrophage RNA Isolation Protocol from Co-Culture System

Following the experimental incubation period, IVD slices were carefully removed using sterile forceps and rinsed well in Eppendorf tubes for flash freezing. The wells were then incubated in TRIzol for 5 minutes at room temperature to collect all cell lysate. After incubation, the TRIzol solution from each well was collected, and any residual agarose gel was removed via brief centrifugation. Microscopic inspection at the end of the co-culture period showed that the majority of macrophages were adherent to the bottom of the well, with minimal presence on the surface of the IVD slices, and negligible infiltration into the agarose matrix. This suggests that the TRIzol incubation was sufficient to recover the majority of macrophage-derived RNA, including any macrophages associated with the tissue surface. To further control for variability in RNA yield between samples, total RNA concentrations were quantified using a Nanodrop spectrophotometer. Equal amounts of RNA (1 µg/sample) were used for cDNA synthesis and subsequent RT-qPCR analysis.

Additional Information on Analysis of PLM Images

Disc tissues were sectioned at a thickness of 10 µm. To assess collagen fiber organization in the annulus fibrosus (AF), PLM images were acquired at ×200 magnification under consistent illumination and polarization settings. Regions of interest (ROIs) were defined as the central AF region. For quantitative analysis, the entire field of view at ×200 magnification was used as the ROI to ensure consistency across samples.

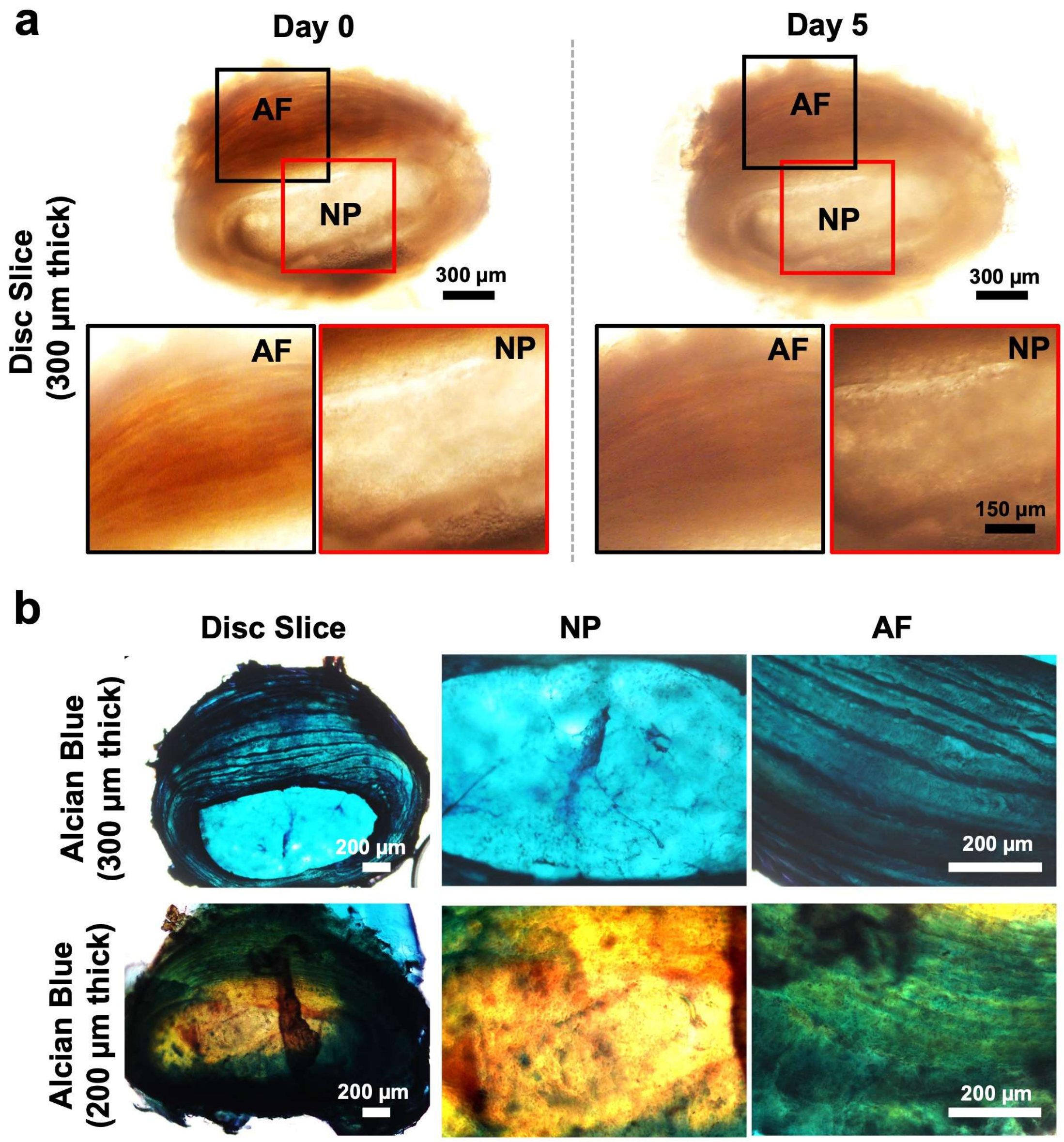

Supplementary Figure S1. The structure of the disc tissue slice is comparable between fresh tissue and day 5 culture. (a) Brightfield images depict a 300 µm disc tissue slice cultured over a 5-day period. AF and NP regions show minimal structural changes up to day 5. **(b)** Optimal slice thickness was determined to be 300 µm. Fresh tissue slices were stained with Alcian Blue and visualized under a microscope. Slices of 200 µm thickness or thinner were difficult to section and often lost structural integrity during the culture. The AF collagen fibers appeared less distinct, with disrupted architecture, and the NP regions displayed increased fragmentation and disorganization. The experiment was repeated at least three times, with six disc slices per time point in each experiment.

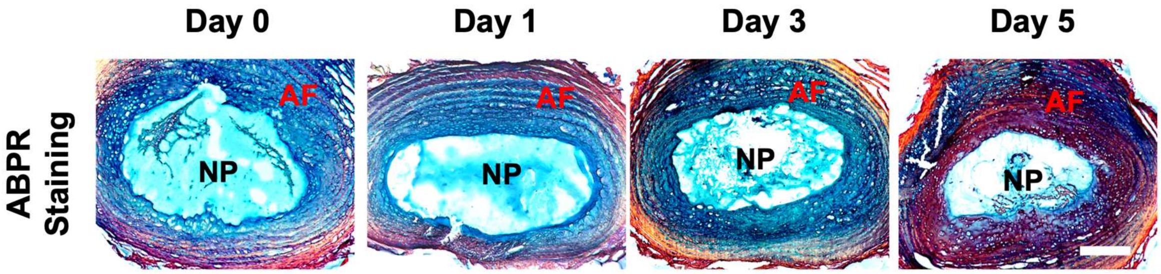

Supplementary Figure S2. Histological images show disc slice structure over the culture period. Alcian Blue/Picrosirius Red (ABPR) staining confirmed the retention of proteoglycans and collagens in both the AF and NP regions. Tissue slices were cultured, fixed, and cryosectioned (10 μm thick). ABPR staining indicated that by day 5, proteoglycan content in the NP and inner AF began to decline, suggesting that day 5 marks a turning point in the disc slice culture. Each staining experiment was independently repeated at least three times. In each experiment, a minimum of three disc slices were used per time point. Scale bar: 200 μm

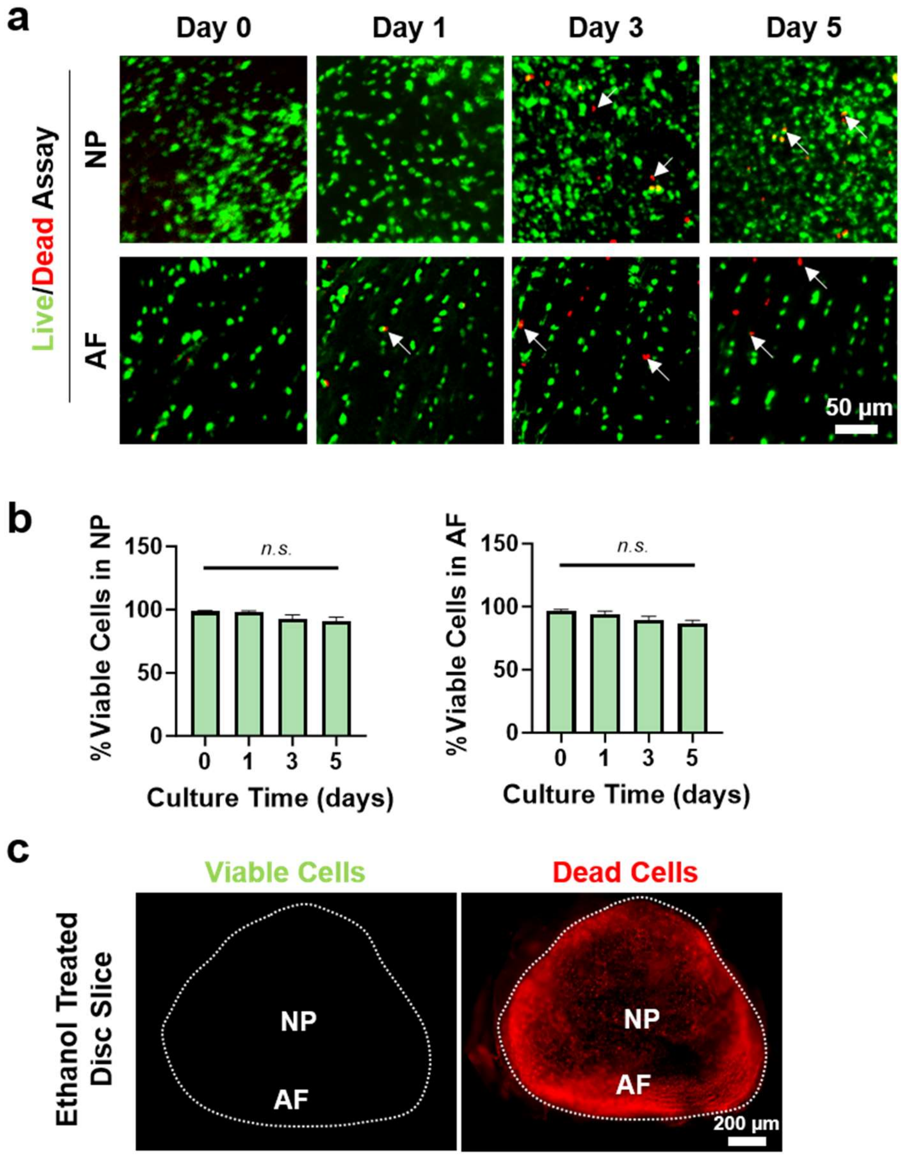

Supplementary Figure S3. Ex vivo culture of whole disc exhibits minimal cell loss for up to 5 days. (a) Representative live/dead images demonstrated a gradual decline in disc cell viability in both the NP and AF over 5 days of *ex vivo* culture. Arrows indicate dead (red) cells. **(b)** Quantitative analysis showed a decreasing trend in the percentage of viable cells in the NP and AF regions over time (~10-20% decline), although the changes did not reach statistical significance. **(c)** Positive control: Disc slices treated with 90% ethanol showed significant cell death. The ethanol-treated disc slices exhibited all dead cells (red), validating the effectiveness of the assay in detecting cell viability. The dotted lines depict the outline of the disc slice. Both conditions were prepared without agarose re-embedding to ensure dye exposure and penetration. The experiment was repeated for three times, with at least three slices used per time point in each experiment.

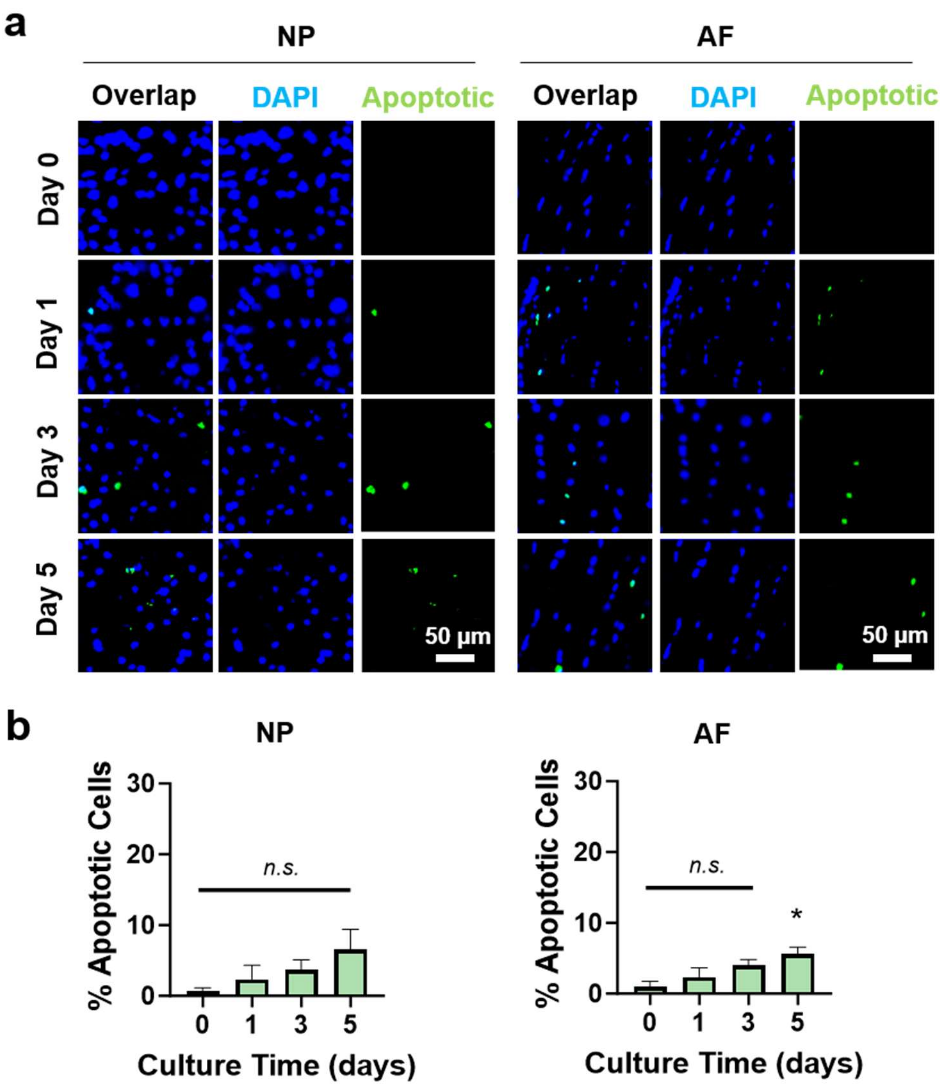

Supplementary Figure S4. Few disc cells underwent apoptosis during disc slice culture. Tissue slices were analyzed using the terminal deoxynucleotidyl transferase-mediated dUTP nick-end labeling (TUNEL) assay to detect apoptotic cells. The percentage of apoptotic cells in both the AF and NP regions remained relatively constant up to day 5. One-way ANOVA with multiple comparisons was performed. Each staining experiment was independently repeated at least three times. For each experiment, a minimum of three disc slices were used per time point. *n.s.,* not significant; *p<0.05 for day 5 vs day 0 in AF.

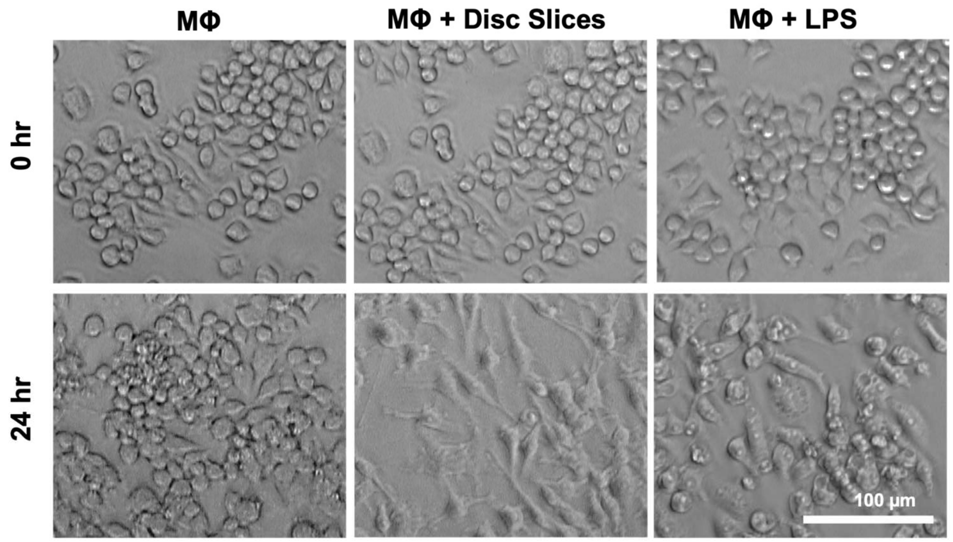

Supplementary Figure S5. Changes are observed in macrophage (MF) morphology following disc tissue slice co-culture. Cell morphology was assessed before and after 24 hours of co-culture with disc slices. Macrophages cultured alone exhibited minimal morphological changes and maintained a rounded shape. In contrast, macrophages co-cultured with disc slices showed a transition from rounded, compact cells to an elongated, spread-out morphology, indicative of macrophage activation. LPS-treated cells served as positive controls and similarly demonstrated morphological changes.

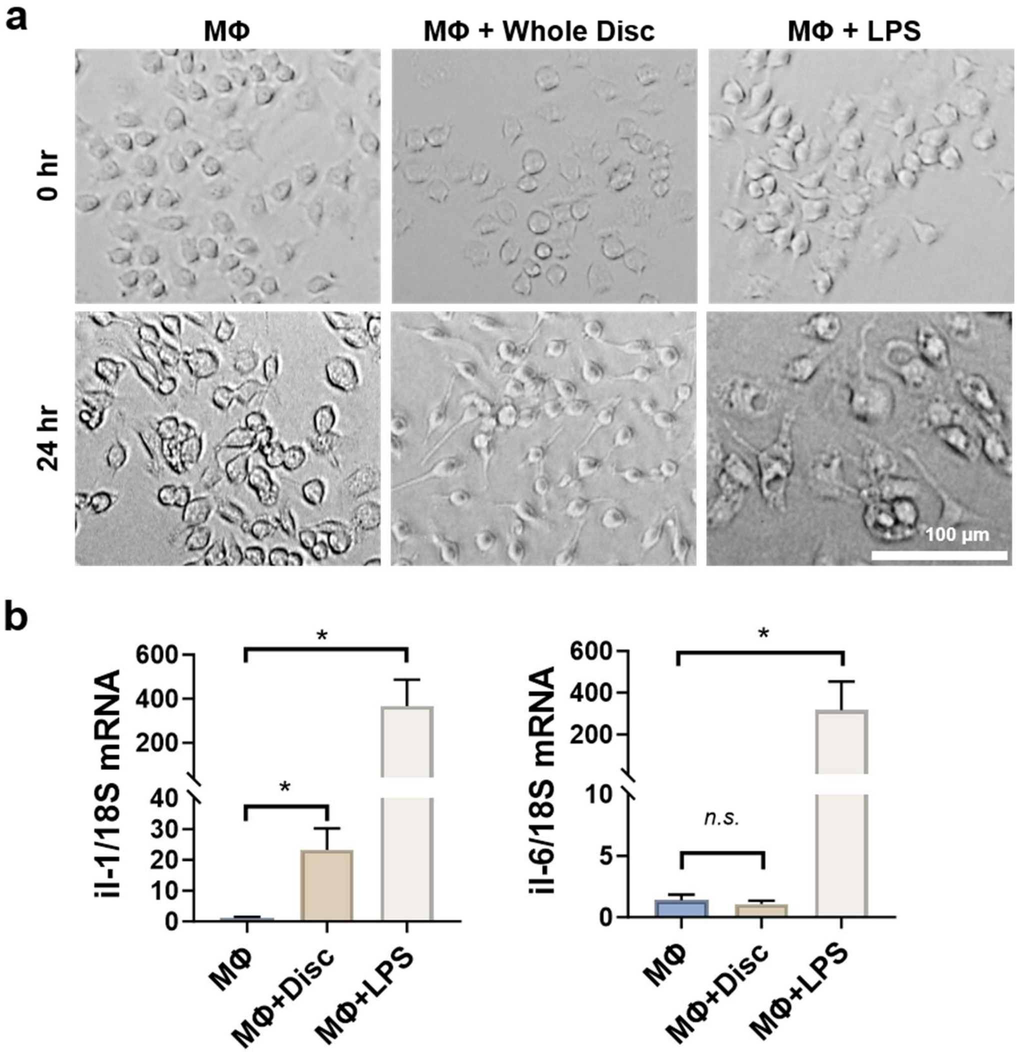

Supplementary Figure S6. Changes are observed in macrophage (MF) morphology following co-culture with whole discs. (a) Cell morphology was evaluated before and after 24 hours of co-culture with intact whole discs. Compared to disc slices, macrophages co-cultured with whole discs exhibited similarly elongated shapes. In both cases, LPS-treated groups showed comparable morphological changes indicative of macrophage activation. **(b)** Gene expression analysis revealed a significant increase in *il-1* in the whole disc co-culture group compared to the control, indicating a pro-inflammatory response. In contrast, *il-6* levels were similar between the macrophage-only and co-culture groups. LPS treatment effectively induced both *il-1* and *il-6* expression. Data were collected from at least three independent experiments. In each experiment, a minimum of three wells were used per condition, with four disc slices in each co-culture well. * p<0.05; *n.s.,* not significant; by one-way ANOVA with multiple comparisons.

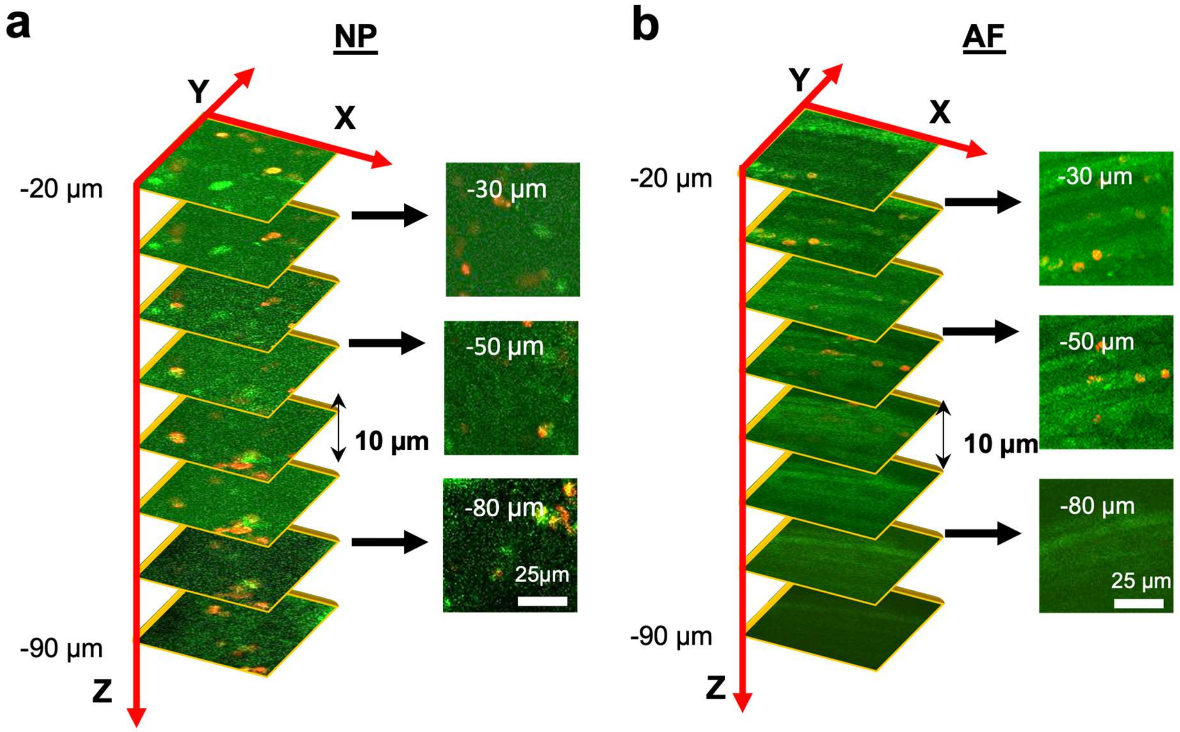

Supplementary Figure S7. Analysis of macrophage penetration was performed on the 3D reconstructed images of disc slices in the NP (a) and AF (b) regions, respectively. Note: Macrophages (red) were counted at each tissue depth (10 µm intervals). The top 20 µm surface layer was excluded from analysis to avoid non-penetrated cells. In the NP, macrophages were observed as deep as 90–110 µm, while in the AF region, most macrophages were located around 50 µm depth. Experiment was repeated at least three times.

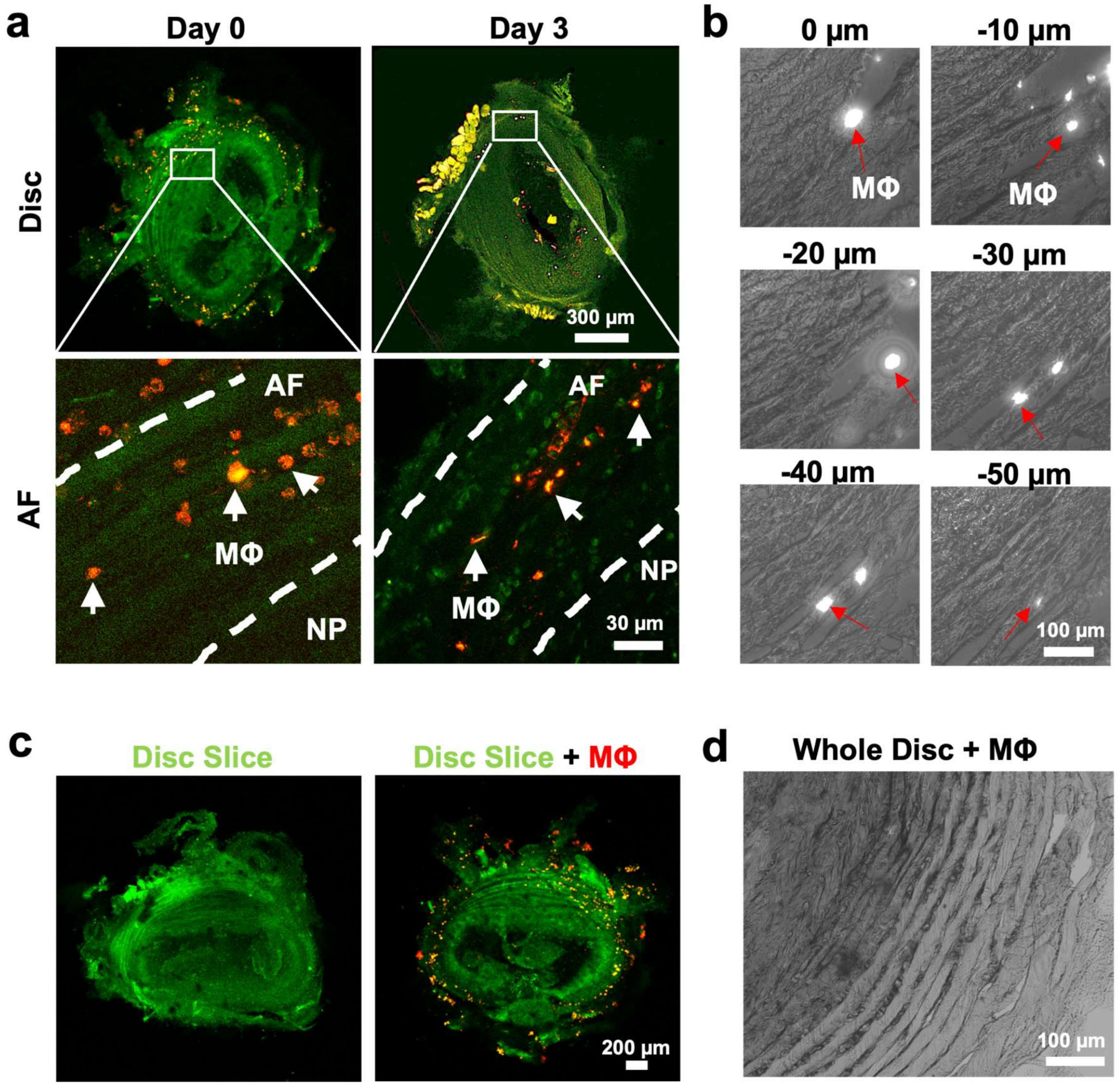

Supplementary Figure S8. Macrophage infiltration and depth were evaluated in disc slices. **(a)** 3D reconstructed fluorescence images of disc slices overlaid with macrophages showed successful macrophage penetration into disc tissue. Comparison of Day 0 and Day 3 disc slices demonstrated macrophage penetration at both time points. White arrows indicate macrophages in disc tissue slice. **(b)** Representative thin cryosections (10 μm) of disc slices showed macrophages penetrating up to 50 μm deep from the tissue surface (0 μm). Red arrows indicate infiltrated macrophages at various tissue depth. **(c)** A negative control of CellTracker™ CM-DiI Dye labeled macrophages confirmed the staining specificity of macrophages used in the study. **(d)** Mid-section of a whole disc tissue overlaid with DiI dye-labeled macrophages showed no macrophage penetration into disc tissue. The experiment was repeated at least three times, with three disc slices per condition in each experiment.

Supplementary Table S1. Primer sequences for real-time RT-PCR.

Target genes	Sequence (5' to 3')
**Mouse 18S**	F: CGGCGACGACCCATTCGAAC<br>R: GAATCGAACCCTGATTCCCCGTC
**Mouse acan**	F: AGGACCTGGTAGTGCGAGTG<br>R: GCGTGTGGCGAAGAA
**Mouse il-6**	F: TTCCATCCAGTTGCCTTCTTG<br>R: TTGGGAGTGGTATCCTCTGTGA
**Mouse adamts4**	F: ACTGGTGGTGGCAGATGACA<br>R: TCACTGTTAGCAGGTAGGGCTTT
**Mouse mmp-13**	F: GGACAAGTAGTTCCAAAGGCTACAA<br>R: CTTTTGCCGGTGTAGGTGTAGATAG
**Mouse col2α1**	F: TCCAGGATCTGCACTGAATG<br>R: TCTGCCCAGTTCAGGTCTCT
**Mouse il1**	F: CATGGAATCCGTGTCTTCCT<br>R: GAGCTGTCTGCTCATTCACG
**Mouse tnf-α**	F: GGCTGCCCCGACTACGT<br>R: GACTTTCTCCTGGTATGAGATAGCAA
**Mouse inos**	F: GTTCTCAGCCCAACAATACAAGA<br>R: GTGGACGGGTCGATGTCAC

Additional Discussion on Macrophage Overlay

Regarding macrophage infiltration, agarose was not applied on top of the tissue slices in the macrophage overlay experiment. Instead, the disc slices were only stabilized around the sides with agarose, leaving the top surface fully exposed for direct contact with macrophages. Therefore, the experiment did not involve agarose encapsulation that would restrict access or alter diffusion. While it is theoretically possible that some macrophages could migrate into the lateral agarose boundary, this is unlikely given the observed infiltration pattern and the experimental design. To further reduce the possibility of non-specific surface attachment, the slices were thoroughly rinsed three times with a large volume of PBS to remove any loosely attached or floating cells prior to imaging. During 3D imaging, the disc surface was used as the starting point for Z-stack acquisition to ensure macrophage localization was assessed within the tissue. Additionally, cryo-sectioning and imaging confirmed that macrophages had penetrated the nucleus pulposus (NP) and annulus fibrosus (AF) regions. Taken together, these steps demonstrate that macrophages infiltrated the intervertebral disc tissue itself, and that the surrounding agarose scaffold had no significant impact on this process.

Additional Discussion on Limitations

For macrophage overlay study, while the current model uses Raw 264.7 macrophages in direct co-culture to study inflammation-induced disc degeneration, it does not capture immune cell trafficking or resolution mechanisms. Additionally, the macrophage source, activation state, and cell ratio can influence inflammatory outcomes. Future efforts will involve primary macrophage populations, polarization studies, and more complex platforms that incorporate immune recruitment dynamics to better approximate in vivo conditions. While our study demonstrates pro-inflammatory macrophage activation and migration, it does not yet assess downstream functional consequences such as phagocytic activity or matrix remodeling. Future experiments will include these assessments to better delineate the role of macrophages in early versus progressive stages of disc degeneration.
